# Neuromuscular Electrical Stimulation as a Potential Countermeasure for Skeletal Muscle Atrophy and Weakness During Human Spaceflight

**DOI:** 10.3389/fphys.2019.01031

**Published:** 2019-08-13

**Authors:** Nicola A. Maffiuletti, David A. Green, Marco Aurelio Vaz, Marlou L. Dirks

**Affiliations:** ^1^Human Performance Lab, Schulthess Clinic, Zurich, Switzerland; ^2^Space Medicine Team, HRE-OM, European Astronaut Centre, European Space Agency, Cologne, Germany; ^3^KBRwyle, Wyle Laboratories GmbH, Cologne, Germany; ^4^King’s College London, Centre for Human & Applied Physiological Sciences (CHAPS), London, United Kingdom; ^5^Exercise Research Laboratory (LAPEX), Federal University of Rio Grande do Sul, Porto Alegre, Brazil; ^6^Department of Sport and Health Sciences, University of Exeter, Exeter, United Kingdom

**Keywords:** muscle atrophy, spaceflight analog, countermeasure, muscle weakness, electrical stimulation

## Abstract

Human spaceflight is associated with a substantial loss of skeletal muscle mass and muscle strength. Neuromuscular electrical stimulation (NMES) evokes involuntary muscle contractions, which have the potential to preserve or restore skeletal muscle mass and neuromuscular function during and/or post spaceflight. This assumption is largely based on evidence from terrestrial disuse/immobilization studies without the use of large exercise equipment that may not be available in spaceflight beyond the International Space Station. In this mini-review we provide an overview of the rationale and evidence for NMES based on the terrestrial state-of-the-art knowledge, compare this to that used in orbit, and in ground-based analogs in order to provide practical recommendations for implementation of NMES in future space missions. Emphasis will be placed on knee extensor and plantar flexor muscles known to be particularly susceptible to deconditioning in space missions.

## Introduction

Prolonged exposure to microgravity is associated with multi-system deconditioning including the cardiovascular (Hargens and Richardson, 2009) and musculoskeletal systems (Narici and de Boer, 2011). For instance, spaceflight-induced decrements in bone mineral density (Vico and Hargens, 2018) and skeletal muscle mass (Fitts et al., 2010) are common, particularly in lower-limb muscles (LeBlanc et al., 1995). Despite the considerable subject variability in the extent of muscle atrophy and functional loss, one of the most affected muscles seems to be the triceps surae, for which muscle fiber atrophy of 20% has been observed after 6 months of spaceflight (Fitts et al., 2000; Fitts et al., 2010). Long-term spaceflight is also known to impair functionality (Mulavara et al., 2018), neuromuscular control (Cohen et al., 2012) and skeletal muscle strength (Tesch et al., 2005; Shiba et al., 2015), with the strength decline primarily reflecting the loss of muscle mass (Fitts et al., 2010). Since the Skylab missions, it has been known that spaceflight induces more weakness in thigh than arm muscles, particularly the knee extensors, for which ∼20% of strength loss was reported after 1- and 2-month missions (Fitts et al., 2000). Recent studies suggest that in some individuals there are persistent neuromuscular control issues - compounded by and/or related to neurovestibular dysfunction (e.g., Van Ombergen et al., 2017) - resulting in extended periods of physical rehabilitation upon return to Earth (Lambrecht et al., 2017; Petersen et al., 2017).

Besides muscle atrophy, spaceflight-related muscle weakness appears also to reflect a number of neuromuscular alterations, including a selective transformation of slow muscle fibers (type I) to faster phenotypes (type II) (Trappe et al., 2009). In fact, there is evidence that slow muscle fibers are predominantly affected by spaceflight (Fitts et al., 2000; Yamakuchi et al., 2000; Sandona et al., 2012; Wang and Pessin, 2013). Recent pilot data from the SARCOLAB study also suggest that reduced plantar flexor muscle volume may be associated with altered muscle architecture, contractile protein composition, and impaired muscle fiber contractility (Rittweger et al., 2018).

### Exercise Training as a Countermeasure

In order to address microgravity-induced deconditioning, exercise countermeasure training is performed daily on the International Space Station (ISS) (Hackney et al., 2015). Despite the medical standard agreements between the ISS international partners, each partner utilizes different training regimes that are to some extent individually tailored for each crewmember. For example, exercise countermeasures in the United States operating segment (NASA, ESA, JAXA, and CSA) consist of an integrated resistance and aerobic training schedule employing the advanced resistive exercise device (ARED), the second generation treadmill (T2), and a cycle ergometer with vibration isolation and stabilization (CEVIS) (Petersen et al., 2016). In contrast, the Russian operating segment employs the 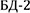 treadmill, the 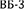 cycle ergometer, and the force loader (HC)-1 installed on the 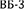 ergometer (Yarmanova et al., 2015). These tools are complemented by a set of resistance bands, compression thigh cuffs, lower body negative pressure trousers, suits for lower body compression and postural (axial) loading and also an electrical stimulator.

Despite the significant investment in both resources and crew time, astronauts typically require a period of rehabilitation upon return to Earth (Lambrecht et al., 2017; Petersen et al., 2017), indicative that deconditioning is not entirely prevented (English et al., 2015; Sibonga et al., 2015). In fact, there appears to be significant variability in the relative effectiveness of ISS countermeasures across various physiological systems (Williams et al., 2009), but also between individuals (Rittweger et al., 2018). The current countermeasure regimes appear unable to fully counteract muscle atrophy and weakness during long-duration ISS missions. For example, even high-volume aerobic training (∼500 km of running) complemented with high-intensity resistance training (∼5000 high-intensity heel raises) were insufficient to prevent plantar flexor weakness and atrophy during a 6-month ISS mission (Rittweger et al., 2018). Furthermore, the current countermeasures require significant time and effort (both for exercise itself and for setup/stowage) in addition to potentially interfering with other crewmember tasks, including experimentation. This explains the increasing attention devoted to consider low-volume, simple and complementary exercise modalities, for use throughout, or potentially for only a short period prior to re-exposure to a gravitational vector, be it Earth, or the hypogravity of the Moon. One of those easily applicable and potentially powerful countermeasures – neuromuscular electrical stimulation (NMES) – is the focus of this article.

### Rationale for NMES

Neuromuscular electrical stimulation involves delivering pre-programmed trains of stimuli to superficial muscles via self-adhesive skin electrodes connected to small portable current generators. Such electrical stimuli can be used to evoke relatively strong (albeit sub-maximal) muscle contractions, whose activation pattern is substantially different from that of voluntary contractions. NMES recruits motor units in a non-selective, spatially fixed, and temporally synchronous pattern (Gregory and Bickel, 2005), with the advantage of activating fast muscle fibers at relatively low force levels, but produces greater muscle fatigue when compared with voluntary actions. If provided repeatedly, NMES improves muscle strength, power and endurance in healthy individuals (Gondin et al., 2011b; Veldman et al., 2016), even though these effects are not superior to those induced by voluntary training (Bax et al., 2005). More importantly, NMES has been shown to preserve/restore muscle mass and aspects of neuromuscular function during/following a period of reduced activity due to illness, injury or surgery (Dirks et al., 2014; Jones et al., 2016; Spector et al., 2016; Maffiuletti et al., 2018), with greater effectiveness compared to other rehabilitation modalities (Bax et al., 2005). As such, NMES is widely used as a rehabilitation strategy for patients with a range of diseases (Jones et al., 2016; Spector et al., 2016), both during and following prolonged physical inactivity. NMES also provides beneficial effects in healthy subjects undergoing short periods of ground-based models of microgravity-induced deconditioning, e.g., bed rest or limb immobilization (Dirks et al., 2014). The majority of terrestrial NMES research has involved stimulation of knee extensor and/or plantar flexor muscles, whose atrophy and weakness can significantly impair locomotion. Although traditional countermeasures have the potential to partially attenuate spaceflight-induced muscle alterations (Fitts et al., 2010), no direct comparison of the effectiveness of these countermeasures versus NMES currently exists.

As such, this mini-review is focused on the use of NMES as a potentially-complementary countermeasure against skeletal muscle atrophy and weakness induced by human spaceflight. We provide an overview of the rationale and evidence for NMES-based terrestrial state-of-the-art knowledge, compare this to that employed in orbit and in ground-based analogs, and provide practical recommendations for possible NMES implementation in future space (or analog) missions.

## NMES in Orbit: Sub-Optimal Use and Evidence

Roscosmos have employed different NMES devices (see top of Table 1) in orbit and in ground-based analogs (Kozlovskaya, 2008). The Tonus-3 unit (Yarmanova et al., 2015) possesses four programs designed to stimulate: calf and quadriceps; calf and hamstring; calf, abdominal and back muscles; and shoulder muscles. Pulses have a duration of 1 ms and maximum current amplitude is ∼300 mA. Stimulation frequency is 10 kHz modulated at 60 Hz. Stimulation (ON) time is 0.5/1.5 s with a non-stimulation (OFF) period of 1.5 s, or alternatively an ON time of 10 ± 1 s with an OFF time of 50 ± 5 s. Another Russian stimulator, the Stimul-01 HF Set, generates high-frequency alternating sinusoidal electrical stimuli at 2.5 kHz with rectangular pulses modulated at 50 Hz. This device is intended for 40-min stimulation periods of lower limb, back, neck, shoulder and arm muscles, although few details have been published (Kozlovskaya et al., 2009). The Stimul-01 LF Set, a wearable NMES system, was uploaded to the ISS in 2006 (Yarmanova et al., 2015) based on data suggesting that low-frequency stimulation is an effective countermeasure against the effects of ground-based (dry immersion) gravitational unloading (Kozlovskaya et al., 2009). The Stimul-01 LF Set provides NMES for 1 s followed by 2 s intervals. The symmetrical bipolar rectangular pulses have a duration of 1 ms and are delivered at 25 Hz, a stimulation pattern considered compatible with work-day activities without being unduly uncomfortable.

**TABLE 1:** In orbit and ground-based analog NMES devices/studies and recommendations for NMES use in spaceflight.

**[Device]/(Study)**	**Disuse model**	**Duration (days)**	**NMES Muscle**	**NMES Duration**	**NMES freq (Hz)**	**NMES on:off (s)**	**NMES Intensity**	***Positive effect on muscle mass?***	***Positive effect on muscle strength?***
[Tonus-3]	Russian Space Station		Multiple muscles		10,000(60)	1.5:1.5 10:50			
[Stimul-01 HF Set]	Russian Space Station		Multiple muscles		2,500(50)				
[Stimul-01 LF Set]	Russian Space Station		Multiple muscles		25	1:2			
Mayr et al., 1999	Space Station (proposal)	/	Lower limbs	6 h/day	25–50	1:2	20–30% max tetanic force	/	/
Shiba et al., 2015	ISS (*n* = 1; ? years)	30 (188-d stay)	Upper arm	47 min/week (3×/week)	?	2:2	80% max comfortable intensity	YES	NO
Gould et al., 1982	Long-leg cast (*n* = 10; 20 years)	14	Lower limbs	16 h/day	37	5:150	Tolerance	YES	∼
Gibson et al., 1988	Long-leg cast (*n* = 7; 26 years)	40	Quad	60 min/day	30	2:9	Visible contraction (5% MVC)	YES	/
Duvoisin et al., 1989	Bed rest (*n* = 3; 36 years)	30	Lower limbs	40 min/day (2×/day)	60	4:16	Tolerance (torque recording)	YES	YES
Dirks et al., 2014	Full-leg cast (*n* = 12; 23 yrs)	5	Quad	80 min/day (2×/day)	100	5:10	Visible palpable full contraction	YES	NO
Reidy et al., 2017	Bed rest (*n* = 10; 70 years)	5 (with proteins)	Quad	120 min/day (3×/day)	75	4:10	Max tolerated intensity	YES	NO
Zange et al., 2017	Unloading device (*n* = 7; 26 years)	60 (with proteins)	Calf	40 min/day (2×/day)	30	5:5	Max tolerated intensity	YES	∼
**Recommendations**			**Quad Calf**	**60 min/day (2x/day)**	**30**	**5:10**	**5 min to reach max tolerated intensity, then increased whenever possible to ≥20% MVC**		

*In gray, in orbit devices/studies (the others are analogs). In bold, recommendations for future implementation of NMES in space. ISS, International Space Station; MVC, maximal voluntary contraction. ∼ = Inconsistent; ? = Unknown.*

Mayr et al. (1999) described an EMG-NMES system (MYOSTIM-FES) embedded into a fabric garment for delivering NMES to the main lower-limb muscle groups (Table 1). The astronaut using this system was reported to be “in a much better condition during flight and after landing” (Freilinger and Mayr, 2002), although no supporting data were published. In another ISS study, NMES was delivered in the final 30 days of a 188-day mission. A 12% increase in triceps brachii muscle volume within a single astronaut was demonstrated, while muscle volume of the non-stimulated triceps was essentially unchanged (Shiba et al., 2015). Whilst limited, these data suggest that NMES application during spaceflight is feasible and potentially able to (at least partially) prevent muscle atrophy. However, currently there is a paucity of data on both NMES effectiveness in orbit, and an evidence-based rationale for its optimal use.

## NMES in Ground-Based Analogs

The major underlying cause of muscle atrophy in microgravity is a net negative muscle protein balance (Phillips et al., 2009; Wall and van Loon, 2013). Given the challenge of experimentation and countermeasures testing in space, ground-based models of microgravity such as tilted head-down bed rest, lower-limb immobilization or axial unloading are generally used. Such models have demonstrated a substantial decrease in postabsorptive and postprandial muscle protein synthesis (Gibson et al., 1987; Ferrando et al., 1996; Biolo et al., 2004; Glover et al., 2008; Wall et al., 2013; Wall et al., 2016), which is suggested to be accompanied by an increase in muscle protein breakdown in the early phase of disuse (Urso et al., 2006; Abadi et al., 2009; Wall et al., 2016). Even if the impact of spaceflight on muscle protein turnover has yet to be investigated, a similar decrease in whole-body protein synthesis was observed following long-term (>3 months) spaceflight (Stein et al., 1999). Although it remains to be established whether the same holds true for muscle (rather than whole-body) protein turnover, countermeasures which stimulate muscle protein synthesis, while simultaneously suppressing muscle protein breakdown, are likely to be effective in partially preventing muscle atrophy during prolonged spaceflight.

Long-duration bed rest induces significant muscle weakness and atrophy (Mulavara et al., 2018). Dry immersion has been shown to elicit rapid and profound losses of lower limb-muscle contractile properties e.g., triceps surae (Kozlovskaya et al., 1984; Koryak, 1998, 1999), similar to those observed in-flight (Koryak, 2001), with signs of muscle denervation appearing after only 3 days (Demangel et al., 2017). The effects of daily low- and high-frequency NMES upon lower-limb muscles were evaluated during 7 days of dry immersion and 105 days of isolation (Koryak et al., 2008; Kozlovskaya, 2008; Koryak, 2018). Low-frequency stimulation was effective in counteracting triceps surae force-velocity property decrements, particularly with high stimulation intensities.

Various NMES protocols have been employed in a range of ground-based analog studies in an attempt to attenuate muscle atrophy and weakness in healthy subjects (Table 1). Despite the diversity in NMES parameters and protocols between studies (ranges for duration: 40 min to 16 h per day; frequency: 30 to 100 Hz; intensity: visible contraction to maximum tolerated current), a common finding is that daily NMES is an effective countermeasure to prevent the loss of lower-limb muscle mass associated to short-term disuse (from 5 to 60 days) as a result of casting (Gould et al., 1982; Gibson et al., 1988; Dirks et al., 2014), bed rest (Duvoisin et al., 1989; Reidy et al., 2017) and axial unloading (Zange et al., 2017). Mechanistically, this is probably due to an increase/maintenance of muscle protein synthesis (Gibson et al., 1988; Wall et al., 2012), but may also be accompanied by a suppression of muscle protein breakdown (Dirks et al., 2014). NMES might also affect other intramuscular processes including (but not limited to) emission of reactive oxygen species, insulin signaling and substrate utilization, but this is outside the scope of this mini-review (Min et al., 2011; Zuo et al., 2011). Whilst NMES preserved muscle strength in one of the analog studies (Duvoisin et al., 1989), the effects have been inconsistent (Table 1). As such, despite the clear potential of NMES to maintain muscle mass during unloading [particularly when complemented with protein supplementation (Dirks et al., 2017)], careful definition of NMES implementation is vital to ensure optimal muscle functional outcomes.

## State-of-Knowledge on Terrestrial NMES

Much work has been conducted to identify the optimal evidence-based NMES parameters/protocols for neuromuscular training/rehabilitation (Maffiuletti, 2010; Maffiuletti et al., 2018). One of the main conclusions is that the externally-controllable parameters (e.g., current and electrode characteristics) have a minor impact on NMES effectiveness (Lieber and Kelly, 1991). In fact, NMES utilization has varied substantially between in orbit, ground-based analog and terrestrial studies (see Table 1), despite calls for standardization as long as 30 years ago (Singer et al., 1987).

There is, however, increasing evidence that NMES effectiveness is proportional to the amount of evoked force/tension (Gondin et al., 2011a). This is generally expressed as a percentage of the maximal voluntary strength, and is referred to as “NMES training intensity.” For example, in Lai et al. (1988) two groups of healthy volunteers had their quadriceps stimulated for 3 weeks at low vs. high NMES training intensities. NMES effectiveness, defined as an improvement in maximal strength mediated by NMES, was linearly related to NMES training intensity (+24 and +48% in respective groups). Therefore, NMES training intensity, not current intensity/subjective current level or any other stimulation parameter, should be (1) considered as the main determinant of NMES effectiveness, (2) quantified whenever possible on an individual basis, and (3) maximized whenever possible by means of multiple subterfuges (see e.g., Maffiuletti, 2010). Methodologically, at least four simple strategies are able to amplify the muscle response to NMES while minimizing the current-induced discomfort (i.e., the main limitation of NMES): (1) localizing the muscle motor point (i.e., the skin area above the stimulated muscle where the motor threshold is the lowest for a given electrical pulse) (Gobbo et al., 2014); (2) implementing a familiarization period of a few days; (3) providing control of the stimulation unit directly to the participant; (4) allowing the participant to contract the stimulated muscle voluntarily to divert attention from pain/discomfort induced by NMES (Maffiuletti, 2010).

## Future NMES Recommendations for Spaceflight

Based on terrestrial best practice we recommend the following approach for possible NMES utilization in future space missions, including long-term exploration (see also bottom of Table 1). NMES should be seen as a complement to, rather than a substitute for pre-existing exercise countermeasures. This implies careful planning of daily exercise training by considering that NMES is performed in static conditions – so that other non-physical tasks can be executed concomitantly – and separately from the other exercise modalities (i.e., NMES is not superimposed to running, cycling or rowing).

### General Settings

Simultaneous bilateral stimulation of quadriceps femoris and triceps surae muscles should be performed using two large rectangular/elliptical electrodes per muscle (one distal and one proximal) using a space-compatible portable 4-channel stimulator. Astronauts should be in a seated position, with knee and ankle joints restrained at 90°. This knee angle is known to reduce the involvement of the biarticular gastrocnemii, which are less susceptible to muscle atrophy than the soleus (Fitts et al., 2010). In this static position, reasonable levels of muscle tension can be generated even in the absence of gravity (tension will be much lower if limb movement is permitted), which is an important prerequisite for maintaining/increasing the force generating capacity of a muscle (Lieber and Kelly, 1993).

### Current-Related Settings

Biphasic sinusoidal/rectangular pulses with a duration of at least 400–600 μs should be used, with an OFF time at least twice the ON time (e.g., 5 s ON:10 s OFF). Stimulation frequency should be close to 30 Hz to ensure full tetanic contractions while minimizing fatigability (Spector et al., 2016).

### Before Spaceflight

Prior to flight, muscle motor points should be localized and marked on the skin according to the methodology proposed by Gobbo and co-workers (Gobbo et al., 2014). A familiarization period of ∼1 week (3–5 short sessions) should be performed to improve tolerance and thus adherence, whilst also minimizing any risk of muscle damage. Critically, the individual current intensity-evoked force relationship should be determined for each stimulated muscle group of each crewmember, which would require the use of a dynamometer (such as the MARES).

### During Spaceflight

Neuromuscular electrical stimulation should ideally be performed twice daily, with stimulation periods of ∼30 min. Current intensity should be progressively increased to the maximally tolerated level during the first 5 min of each session, to ensure strong muscle contractions. Current intensity should thereafter be increased throughout the entire session, ideally whenever possible. At the end of each session, average current intensity (per channel/muscle), discomfort (0–10 scale) and maximal evoked force (if available) should be recorded. NMES should preferably be combined with protein ingestion to augment its effect on muscle mass (Dirks et al., 2017).

## Remaining NMES Space Challenges

Whilst NMES-based resistance training has potential as an inflight countermeasure, it has some limitations such as the inability to activate the entire muscle (Maffiuletti, 2010), issues with dose and tolerance (discomfort) but no long-term safety concerns (Maffiuletti et al., 2018), and unclear effects upon other physiological systems known to decondition in space (e.g., skeletal or cardiovascular systems). Nevertheless, knowledge of the methodological and physiological specificities of NMES would allow end-users to optimally apply NMES as a complement to other countermeasures for preserving lower-limb functionality (Maffiuletti, 2010).

This mini-review has focused upon muscles acting around the knee and ankle joints. Nevertheless, other muscle groups such as back extensors (Chang et al., 2016) have been shown to decondition in space, leading to functional and operational consequences (Green and Scott, 2017). Whilst NMES has recently been used upon other muscle groups and multiple body segments simultaneously (Kemmler et al., 2010), as well as with agonist-antagonist co-contraction (Shiba et al., 2015), these modalities appear unsuitable at this stage because of practical considerations and lack of convincing evidence.

## Conclusion

Due to the significant discrepancies between the terrestrial (clinical and experimental) NMES state-of-the-art, and that currently performed in orbit and in analog studies, it is crucial to use optimal NMES knowledge on Earth to revisit and further develop NMES as a feasible strategy for human spaceflight exploration.

## Author Contributions

All authors conceived the manuscript, wrote, reviewed, and approved the final version of the manuscript.

## Conflict of Interest Statement

DG was employed by Wyle Laboratories GmbH but the work and its recommendations were unaffected by any commercial or financial interests. The remaining authors declare that the research was conducted in the absence of any commercial or financial relationships that could be construed as a potential conflict of interest.

## References

[B1] Abadi A. Glover E. I. Isfort R. J. Raha S. Safdar A. Yasuda N. (2009). Limb immobilization induces a coordinate down-regulation of mitochondrial and other metabolic pathways in men and women. *PLoS One* 4:e6518. 10.1371/journal.pone.0006518 19654872 PMC2716517

[B2] Bax L. Staes F. Verhagen A. (2005). Does neuromuscular electrical stimulation strengthen the quadriceps femoris? A systematic review of randomised controlled trials. *Sports Med.* 35 191–212. 10.2165/00007256-200535030-00002 15730336

[B3] Biolo G. Ciocchi B. Lebenstedt M. Barazzoni R. Zanetti M. Platen P. (2004). Short-term bed rest impairs amino acid-induced protein anabolism in humans. *J. Physiol.* 558 381–388. 10.1113/jphysiol.2004.066365 15131238 PMC1664959

[B4] Chang D. G. Healey R. M. Snyder A. J. Sayson J. V. Macias B. R. Coughlin D. G. (2016). Lumbar spine paraspinal muscle and intervertebral disc height changes in astronauts after long-duration spaceflight on the international space station. *Spine* 41 1917–1924. 10.1097/brs.0000000000001873 27779600 PMC5588025

[B5] Cohen H. S. Kimball K. T. Mulavara A. P. Bloomberg J. J. Paloski W. H. (2012). Posturography and locomotor tests of dynamic balance after long-duration spaceflight. *J. Vestib. Res.* 22 191–196. 10.3233/VES-2012-0456 23142833 PMC8080311

[B6] Demangel R. Treffel L. Py G. Brioche T. Pagano A. F. Bareille M. P. (2017). Early structural and functional signature of 3-day human skeletal muscle disuse using the dry immersion model. *J. Physiol.* 595 4301–4315. 10.1113/JP273895 28326563 PMC5491890

[B7] Dirks M. L. Groen B. B. Franssen R. Van Kranenburg J. Van Loon L. J. (2017). Neuromuscular electrical stimulation prior to presleep protein feeding stimulates the use of protein-derived amino acids for overnight muscle protein synthesis. *J. Appl. Physiol.* 122 20–27. 10.1152/japplphysiol.00331.2016 27789768

[B8] Dirks M. L. Wall B. T. Snijders T. Ottenbros C. L. Verdijk L. B. Van Loon L. J. (2014). Neuromuscular electrical stimulation prevents muscle disuse atrophy during leg immobilization in humans. *Acta Physiol.* 210 628–641. 10.1111/apha.12200 24251881

[B9] Duvoisin M. R. Convertino V. A. Buchanan P. Gollnick P. D. Dudley G. A. (1989). Characteristics and preliminary observations of the influence of electromyostimulation on the size and function of human skeletal muscle during 30 days of simulated microgravity. *Aviat. Space Environ. Med.* 60 671–678. 2764851

[B10] English K. L. Lee S. M. C. Loehr J. A. Ploutz-Snyder R. J. Ploutz-Snyder L. L. (2015). Isokinetic strength changes following long-duration spaceflight on the ISS. *Aerosp. Med. Hum. Perform.* 86 A68–A77. 10.3357/AMHP.EC09.2015 26630197

[B11] Ferrando A. A. Lane H. W. Stuart C. A. Davis-Street J. Wolfe R. R. (1996). Prolonged bed rest decreases skeletal muscle and whole body protein synthesis. *Am. J. Physiol.* 270 E627–E633. 8928769 10.1152/ajpendo.1996.270.4.E627

[B12] Fitts R. H. Riley D. R. Widrick J. J. (2000). Physiology of a microgravity environment invited review: microgravity and skeletal muscle. *J. Appl. Physiol.* 89 823–839. 10.1152/jappl.2000.89.2.823 10926670

[B13] Fitts R. H. Trappe S. W. Costill D. L. Gallagher P. M. Creer A. C. Colloton P. A. (2010). Prolonged space flight-induced alterations in the structure and function of human skeletal muscle fibres. *J. Physiol.* 588 3567–3592. 10.1113/jphysiol.2010.188508 20660569 PMC2988519

[B14] Freilinger G. Mayr W. (2002). “Electrical stimulation as a countermeasure to muscle alteration in space,” in *Proceedings of the 23rd Annual International Gravitational Physiology Meeting*, Stockholm. 15002597

[B15] Gibson J. N. Halliday D. Morrison W. L. Stoward P. J. Hornsby G. A. Watt P. W. (1987). Decrease in human quadriceps muscle protein turnover consequent upon leg immobilization. *Clin. Sci.* 72 503–509. 10.1042/cs0720503 2435445

[B16] Gibson J. N. Smith K. Rennie M. J. (1988). Prevention of disuse muscle atrophy by means of electrical stimulation: maintenance of protein synthesis. *Lancet* 2 767–770. 10.1016/s0140-6736(88)92417-8 2901612

[B17] Glover E. I. Phillips S. M. Oates B. R. Tang J. E. Tarnopolsky M. A. Selby A. (2008). Immobilization induces anabolic resistance in human myofibrillar protein synthesis with low and high dose amino acid infusion. *J. Physiol.* 586 6049–6061. 10.1113/jphysiol.2008.160333 18955382 PMC2655417

[B18] Gobbo M. Maffiuletti N. A. Orizio C. Minetto M. A. (2014). Muscle motor point identification is essential for optimizing neuromuscular electrical stimulation use. *J. Neuroeng. Rehabil.* 11:17. 10.1186/1743-0003-11-17 24568180 PMC3938308

[B19] Gondin J. Brocca L. Bellinzona E. D’antona G. Maffiuletti N. A. Miotti D. (2011a). Neuromuscular electrical stimulation training induces atypical adaptations of the human skeletal muscle phenotype: a functional and proteomic analysis. *J. Appl. Physiol.* 110 433–450. 10.1152/japplphysiol.00914.2010 21127206

[B20] Gondin J. Cozzone P. J. Bendahan D. (2011b). Is high-frequency neuromuscular electrical stimulation a suitable tool for muscle performance improvement in both healthy humans and athletes? *Eur. J. Appl. Physiol.* 111 2473–2487. 10.1007/s00421-011-2101-2 21909714

[B21] Gould N. Donnermeyer D. Pope M. Ashikaga T. (1982). Transcutaneous muscle stimulation as a method to retard disuse atrophy. *Clin. Orthop. Relat. Res.* 164, 215–220. 6978224

[B22] Green D. A. Scott J. P. R. (2017). Spinal health during unloading and reloading associated with spaceflight. *Front. Physiol.* 8:1126. 10.3389/fphys.2017.01126 29403389 PMC5778142

[B23] Gregory C. M. Bickel C. S. (2005). Recruitment patterns in human skeletal muscle during electrical stimulation. *Phys. Ther.* 85 358–364. 15794706

[B24] Hackney K. J. Scott J. M. Hanson A. M. English K. L. Downs M. E. Ploutz-Snyder L. L. (2015). The Astronaut-Athlete: optimizing human performance in space. *J. Strength Cond. Res.* 29 3531–3545. 10.1519/JSC.0000000000001191 26595138

[B25] Hargens A. R. Richardson S. (2009). Cardiovascular adaptations, fluid shifts, and countermeasures related to space flight. *Respir. Physiol. Neurobiol.* 169(Suppl. 1), S30–S33. 10.1016/j.resp.2009.07.005 19615471

[B26] Jones S. Man W. D. Gao W. Higginson I. J. Wilcock A. Maddocks M. (2016). Neuromuscular electrical stimulation for muscle weakness in adults with advanced disease. *Cochrane Database Syst. Rev.* 10:CD009419. 27748503 10.1002/14651858.CD009419.pub3 PMC6464134

[B27] Kemmler W. Schliffka R. Mayhew J. L. Von Stengel S. (2010). Effects of whole-body electromyostimulation on resting metabolic rate, body composition, and maximum strength in postmenopausal women: the training and electrostimulation trial. *J. Strength Cond. Res.* 24 1880–1887. 10.1519/JSC.0b013e3181ddaeee 20555279

[B28] Koryak Y. (1998). Electromyographic study of the contractile and electrical properties of the human triceps surae muscle in a simulated microgravity environment. *J. Physiol.* 510(Pt 1), 287–295. 10.1111/j.1469-7793.1998.287bz.x 9625885 PMC2231033

[B29] Koryak Y. (1999). Electrical and contractile parameters of muscle in man: effects of 7-day “dry” water immersion. *Aviat. Space Environ. Med.* 70 459–464. 10332940

[B30] Koryak Y. A. (2018). Neuromuscular electrical stimulation in conditions of gravitational unloading. *Sci. Eur.* 23 3–10. 18785352

[B31] Koryak Y. A. Kozlovskaya I. B. Khimoroda N. N. (2008). Low-frequency neuromuscular electrical stimulation training of human skeletal muscles in conditions of gravitational unloading. *Eur. J. Nat. Hist.* 1 86–90.

[B32] Koryak Y. U. (2001). Electrically evoked and voluntary properties of the human triceps surae muscle: effects of long-term spaceflights. *Acta Physiol. Pharmacol. Bulg.* 26 21–27. 11693395

[B33] Kozlovskaya I. B. (2008). Fundamental and applied objectives of investigations in immersion. *Aviakosm Ekol. Med.* 42 3–7. 19192530

[B34] Kozlovskaya I. B. Grigoriev H. P. Gevlich A. I. (1984). Comparative analysis of the influences of weightlessness and its models on speed-strength properties and skeletal muscle tone. *Russian Biol. Aerosp. Med.* 6 22–25.

[B35] Kozlovskaya I. B. Yarmanova E. N. Vinogradova O. L. Shipov A. A. Tomilovskaya E. S. Fomina E. V. (2009). Prospects for using an exercise device to support and rehabilitate the condition of the musculature in various professional and age groups of the population. *Teoriya i Praktika Fizicheskoi Kultury* 3 18–20.

[B36] Lai H. S. Domenico G. D. Strauss G. R. (1988). The effect of different electro-motor stimulation training intensities on strength improvement. *Aust. J. Physiother.* 34 151–164. 10.1016/S0004-9514(14)60607-3 25026069

[B37] Lambrecht G. Petersen N. Weerts G. Pruett C. Evetts S. Stokes M. (2017). The role of physiotherapy in the European space agency strategy for preparation and reconditioning of astronauts before and after long duration space flight. *Musculoskelet. Sci. Pract.* 27(Suppl. 1), S15–S22. 10.1016/j.math.2016.10.009 28173928

[B38] LeBlanc A. Rowe R. Schneider V. Evans H. Hedrick T. (1995). Regional muscle loss after short duration spaceflight. *Aviat. Space Environ. Med.* 66 1151–1154. 8747608

[B39] Lieber R. L. Kelly M. J. (1991). Factors influencing quadriceps femoris muscle torque using transcutaneous neuromuscular electrical stimulation. *Phys. Ther.* 71 715–721. 10.1093/ptj/71.10.715 1946610

[B40] Lieber R. L. Kelly M. J. (1993). Torque history of electrically stimulated human quadriceps: implications for stimulation therapy. *J. Orthop. Res.* 11 131–141. 10.1002/jor.1100110115 8423515

[B41] Maffiuletti N. A. (2010). Physiological and methodological considerations for the use of neuromuscular electrical stimulation. *Eur. J. Appl. Physiol.* 110 223–234. 10.1007/s00421-010-1502-y 20473619

[B42] Maffiuletti N. A. Gondin J. Place N. Stevens-Lapsley J. Vivodtzev I. Minetto M. A. (2018). Clinical use of neuromuscular electrical stimulation for neuromuscular rehabilitation: what are we overlooking? *Arch. Phys. Med. Rehabil.* 99 806–812. 10.1016/j.apmr.2017.10.028 29233625

[B43] Mayr W. Bijak M. Girsch W. Hofer C. Lanmuller H. Rafolt D. (1999). MYOSTIM-FES to prevent muscle atrophy in microgravity and bed rest: preliminary report. *Artif. Organs.* 23 428–431. 10.1046/j.1525-1594.1999.06371.x 10378935

[B44] Min K. Smuder A. J. Kwon O. S. Kavazis A. N. Szeto H. H. Powers S. K. (2011). Mitochondrial-targeted antioxidants protect skeletal muscle against immobilization-induced muscle atrophy. *J. Appl. Physiol.* 111 1459–1466. 10.1152/japplphysiol.00591.2011 21817113 PMC3220313

[B45] Mulavara A. P. Peters B. T. Miller C. A. Kofman I. S. Reschke M. F. Taylor L. C. (2018). Physiological and functional alterations after spaceflight and bed rest. *Med. Sci. Sports Exerc.* 50 1961–1980. 10.1249/MSS.0000000000001615 29620686 PMC6133205

[B46] Narici M. V. de Boer M. D. (2011). Disuse of the musculo-skeletal system in space and on earth. *Eur. J. Appl. Physiol.* 111 403–420. 10.1007/s00421-010-1556-x 20617334

[B47] Petersen N. Jaekel P. Rosenberger A. Weber T. Scott J. Castrucci F. (2016). Exercise in space: the European space agency approach to in-flight exercise countermeasures for long-duration missions on ISS. *Extrem. Physiol. Med.* 5:9. 10.1186/s13728-016-0050-4 27489615 PMC4971634

[B48] Petersen N. Lambrecht G. Scott J. Hirsch N. Stokes M. Mester J. (2017). Postflight reconditioning for European astronauts - A case report of recovery after six months in space. *Musculoskelet. Sci. Pract.* 27(Suppl. 1), S23–S31. 10.1016/j.msksp.2016.12.010 28173929

[B49] Phillips S. M. Glover E. I. Rennie M. J. (2009). Alterations of protein turnover underlying disuse atrophy in human skeletal muscle. *J. Appl. Physiol.* 107 645–654. 10.1152/japplphysiol.00452.2009 19608931

[B50] Reidy P. T. Mckenzie A. I. Brunker P. Nelson D. S. Barrows K. M. Supiano M. (2017). Neuromuscular electrical stimulation combined with protein ingestion preserves thigh muscle mass but not muscle function in healthy older adults during 5 days of bed rest. *Rejuvenation Res.* 20 449–461. 10.1089/rej.2017.1942 28482746 PMC5731550

[B51] Rittweger J. Albracht K. Fluck M. Ruoss S. Brocca L. Longa E. (2018). Sarcolab pilot study into skeletal muscle’s adaptation to long-term spaceflight. *NPJ Microgravity* 4:18. 10.1038/s41526-018-0052-1 30246141 PMC6141586

[B52] Sandona D. Desaphy J. F. Camerino G. M. Bianchini E. Ciciliot S. Danieli-Betto D. (2012). Adaptation of mouse skeletal muscle to long-term microgravity in the MDS mission. *PLoS One* 7:e33232. 10.1371/journal.pone.0033232 22470446 PMC3314659

[B53] Shiba N. Matsuse H. Takano Y. Yoshimitsu K. Omoto M. Hashida R. (2015). Electrically stimulated antagonist muscle contraction increased muscle mass and bone mineral density of one astronaut - initial verification on the international space station. *PLoS One* 10:e0134736. 10.1371/journal.pone.0134736 26296204 PMC4546678

[B54] Sibonga J. D. Spector E. R. Johnston S. L. Tarver W. J. (2015). Evaluating bone loss in ISS astronauts. *Aerosp. Med. Hum. Perform.* 86 A38–A44. 10.3357/AMHP.EC06.2015 26630194

[B55] Singer K. P. De Domenico G. Strauss G. (1987). Electro-motor stimulation research methodology and reporting: a need for standardization. *Aust. J. Physiother.* 33 43–48. 10.1016/S0004-9514(14)60583-3 25025459

[B56] Spector P. Laufer Y. Elboim Gabyzon M. Kittelson A. Stevens Lapsley J. Maffiuletti N. A. (2016). Neuromuscular electrical stimulation therapy to restore quadriceps muscle function in patients after orthopaedic surgery: a novel structured approach. *J. Bone. Joint Surg. Am.* 98 2017–2024. 10.2106/jbjs.16.00192 27926683

[B57] Stein T. P. Leskiw M. J. Schluter M. D. Donaldson M. R. Larina I. (1999). Protein kinetics during and after long-duration spaceflight on MIR. *Am. J. Physiol.* 276 E1014–E1021. 10362613 10.1152/ajpendo.1999.276.6.e1014

[B58] Tesch P. A. Berg H. E. Bring D. Evans H. J. Leblanc A. D. (2005). Effects of 17-day spaceflight on knee extensor muscle function and size. *Eur. J. Appl. Physiol.* 93 463–468. 10.1007/s00421-004-1236-9 15517339

[B59] Trappe S. Costill D. Gallagher P. Creer A. Peters J. R. Evans H. (2009). Exercise in space: human skeletal muscle after 6 months aboard the International space station. *J. Appl. Physiol.* 106 1159–1168. 10.1152/japplphysiol.91578.2008 19150852

[B60] Urso M. L. Scrimgeour A. G. Chen Y. W. Thompson P. D. Clarkson P. M. (2006). Analysis of human skeletal muscle after 48 h immobilization reveals alterations in mRNA and protein for extracellular matrix components. *J. Appl. Physiol.* 101 1136–1148. 10.1152/japplphysiol.00180.2006 16763108

[B61] Van Ombergen A. Demertzi A. Tomilovskaya E. Jeurissen B. Sijbers J. Kozlovskaya I. B. (2017). The effect of spaceflight and microgravity on the human brain. *J. Neurol.* 264 18–22. 28271409 10.1007/s00415-017-8427-x PMC5610662

[B62] Veldman M. P. Gondin J. Place N. Maffiuletti N. A. (2016). Effects of neuromuscular electrical stimulation training on endurance performance. *Front. Physiol.* 7:544 10.3389/fphys.2016.00544 PMC5110544 27899898

[B63] Vico L. Hargens A. (2018). Skeletal changes during and after spaceflight. *Nat. Rev. Rheumatol.* 14 229–245. 10.1038/nrrheum.2018.37 29559713

[B64] Wall B. T. Dirks M. L. Snijders T. Van Dijk J. W. Fritsch M. Verdijk L. B. (2016). Short-term muscle disuse lowers myofibrillar protein synthesis rates and induces anabolic resistance to protein ingestion. *Am. J. Physiol. Endocrinol. Metab.* 310 E137–E147. 10.1152/ajpendo.00227.2015 26578714

[B65] Wall B. T. Dirks M. L. Verdijk L. B. Snijders T. Hansen D. Vranckx P. (2012). Neuromuscular electrical stimulation increases muscle protein synthesis in elderly type 2 diabetic men. *Am. J. Physiol. Endocrinol. Metab.* 303 E614–E623. 10.1152/ajpendo.00138.2012 22739107

[B66] Wall B. T. Snijders T. Senden J. M. Ottenbros C. L. Gijsen A. P. Verdijk L. B. (2013). Disuse impairs the muscle protein synthetic response to protein ingestion in healthy men. *J. Clin. Endocrinol. Metab.* 98 4872–4881. 10.1210/jc.2013-2098 24108315

[B67] Wall B. T. van Loon L. J. (2013). Nutritional strategies to attenuate muscle disuse atrophy. *Nutr. Rev.* 71 195–208. 10.1111/nure.12019 23550781

[B68] Wang Y. Pessin J. E. (2013). Mechanisms for fiber-type specificity of skeletal muscle atrophy. *Curr. Opin. Clin. Nutr. Metab. Care* 16 243–250. 10.1097/MCO.0b013e328360272d 23493017 PMC4327989

[B69] Williams D. Kuipers A. Mukai C. Thirsk R. (2009). Acclimation during space flight: effects on human physiology. *CMAJ* 180 1317–1323. 10.1503/cmaj.090628 19509005 PMC2696527

[B70] Yamakuchi M. Higuchi I. Masuda S. Ohira Y. Kubo T. Kato Y. (2000). Type I muscle atrophy caused by microgravity-induced decrease of myocyte enhancer factor 2C (MEF2C) protein expression. *FEBS Lett.* 477 135–140. 10.1016/s0014-5793(00)01715-4 10899324

[B71] Yarmanova E. N. Kozlovskaya I. B. Khimoroda N. N. Fomina E. V. (2015). Evolution of russian microgravity countermeasures. *Aerosp. Med. Hum. Perform.* 86 A32–A37. 10.3357/AMHP.EC05.2015 26630193

[B72] Zange J. Schopen K. Albracht K. Gerlach D. A. Frings-Meuthen P. Maffiuletti N. A. (2017). Using the Hephaistos orthotic device to study countermeasure effectiveness of neuromuscular electrical stimulation and dietary lupin protein supplementation, a randomised controlled trial. *PLoS One* 12:e0171562. 10.1371/journal.pone.0171562 28207840 PMC5313207

[B73] Zuo L. Nogueira L. Hogan M. C. (2011). Reactive oxygen species formation during tetanic contractions in single isolated Xenopus myofibers. *J. Appl. Physiol.* 111 898–904. 10.1152/japplphysiol.00398.2011 21700897 PMC3174785

